# Model-Based Inference of Synaptic Transmission

**DOI:** 10.3389/fnsyn.2019.00021

**Published:** 2019-08-20

**Authors:** Ola Bykowska, Camille Gontier, Anne-Lene Sax, David W. Jia, Milton Llera Montero, Alex D. Bird, Conor Houghton, Jean-Pascal Pfister, Rui Ponte Costa

**Affiliations:** ^1^Computational Neuroscience Unit, Department of Computer Science, SCEEM, Faculty of Engineering, University of Bristol, Bristol, United Kingdom; ^2^Department of Physiology, University of Bern, Bern, Switzerland; ^3^Department of Physiology, Anatomy and Genetics, Centre for Neural Circuits and Behaviour, University of Oxford, Oxford, United Kingdom; ^4^School of Psychological Science, Faculty of Life Sciences, University of Bristol, Bristol, United Kingdom; ^5^Ernst Strungmann Institute for Neuroscience in Cooperation With Max Planck Society, Frankfurt, Germany; ^6^Frankfurt Institute for Advanced Studies, Frankfurt, Germany; ^7^Institute of Neuroinformatics and Neuroscience Center Zurich, University of Zurich/ETH Zurich, Zurich, Switzerland

**Keywords:** synaptic transmission, short-term synaptic plasticity, model inference, probabilistic inference, quantal analysis

## Abstract

Synaptic computation is believed to underlie many forms of animal behavior. A correct identification of synaptic transmission properties is thus crucial for a better understanding of how the brain processes information, stores memories and learns. Recently, a number of new statistical methods for inferring synaptic transmission parameters have been introduced. Here we review and contrast these developments, with a focus on methods aimed at inferring both synaptic release statistics and synaptic dynamics. Furthermore, based on recent proposals we discuss how such methods can be applied to data across different levels of investigation: from intracellular paired experiments to *in vivo* network-wide recordings. Overall, these developments open the window to reliably estimating synaptic parameters in behaving animals.

## 1. Introduction

Modifications of synaptic transmission properties are believed to underlie learning, memory and, more generally, neural dynamics (Nabavi et al., 2014; Costa et al., 2017; Roelfsema and Holtmaat, 2018; Williams and Holtmaat, 2018; Llera-Montero et al., 2019). It is therefore of great importance to accurately infer synaptic transmission properties. Two key features that define synaptic communication are: stochastic transmission (Malagon et al., 2016) and (relatively fast) temporal dynamics (Markram et al., 1998; Zucker and Regehr, 2002). The former is reflected as trial to trial variability of synaptic transmission as the combined result of pre- and postsynaptic sources of noise, such as probabilistic vesicle release (presynaptic) or binding of quantal neurotransmitter packets to (postsynaptic) receptors (Faber and Korn, 1991; Traynelis et al., 1993). Whereas temporal dynamics is reflected in the temporal modulation of synaptic responses, which is mediated by the multiple time constants of the synaptic transmission machinery. Such dynamics give rise to the commonly observed phenomenon of short-term plasticity (STP) (Tsodyks and Markram, 1997; Zucker and Regehr, 2002). In this review we summarize, discuss and contrast recent developments in inference methods that capture either of these two elements (i.e., stochastic release and STP), or both. In particular our review focus on relatively simple phenomenological and statistical models, which abstract out the underlying biophysics and do not capture some aspects of synaptic transmission.

We also highlight recent advances toward inferring synaptic properties *in vivo*. Studying synaptic transmission parameters under naturalistic conditions is not only likely to give more precise parameters estimates, but also insights into what synaptic transmission properties are relevant in behaving animals (Dobrunz and Stevens, 1999; Isaac et al., 2009).

## 2. Inference of Stochastic Transmission

**Figure 1:**
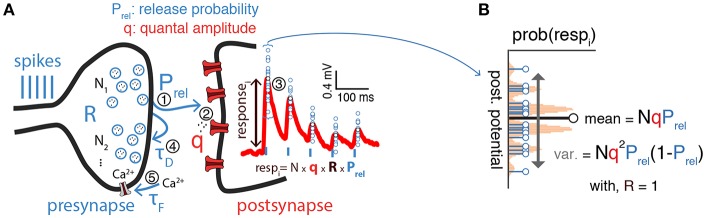
Inference of synaptic transmission parameters. **(A)** Schematic of synaptic transmission parameters. On the left the different elements of the synaptic transmission process are represented: first (1), presynaptic spikes (blue vertical bars) lead to release of vesicles containing neurotransmitter (*R*, for presynaptic resources) from one of *N* possible release sites with probability *P*_rel_; second (2), released neurotransmitters (quanta) bind to postsynaptic receptors triggering a response with amplitude *q*; third (3), this process triggers a postsynaptic response with average amplitude *NqRP*_rel_, which takes into account both binomial and short-term synaptic plasticity; fourth (4), presynaptic vesicles are recovered with a time constant τ_*D*_ which may lead to *short-term depression* of consecutive postsynaptic responses (red trace on the postsynapse) before the presynaptic resources, *R*, fully recover; fifth (5), at the same time presynaptic voltage-dependent calcium (*Ca*^2+^) channels can lead to calcium build-up on the presynapse (modeled by a time constant τ_*F*_), which may increase release probability (*P*_rel_) and in turn lead to an increase of consecutive postsynaptic responses, also known as *short-term facilitation* (not shown). **(B)** Postsynaptic responses exhibit variability [blue circles from **(A)** overlaid on top of the mean postsynaptic response in red]. Such variability is often described as a simple binomial process, with *N* release sites and variance given by $Nq^{2}P_{\text{rel}}\left(1-P_{\text{rel}}\right)$. Plot represents a binomial release model with *N* = 5, *P*_rel_ = 0.5 and some arbitrary *q*.

Synaptic transmission is inherently stochastic (see Figure 1 for a schematic). In the quantal view of synaptic transmission neurotransmitter-containing vesicles (quanta) are released into the synaptic cleft from *N* release sites with probability *P*_rel_ (Del Castillo and Katz, 1954; Korn and Faber, 1991; Larkman et al., 1991; Lanore and Silver, 2016) (Figure 1A). Once released, neurotransmitters bind to postsynaptic receptors triggering a postsynaptic response with mean quantal amplitude *q*. A *binomial model* is often used to describe these three aspects (i.e., number of release sites *N*, release probability *P*_rel_ and the mean quantal amplitude *q*). In this model the mean peak of postsynaptic responses is given by μ = *qNP*_rel_ and their variance by $\sigma^{2}=q^{2}NP_{\text{rel}}\left(1-P_{\text{rel}}\right)$ (Figure 1B)^1^. Several methods based on the binomial release model have been proposed to infer synaptic transmission parameters. A simple method relies solely on using the mean and variance to get estimates of both *q* and *P*_rel_ by rearranging the terms as $\hat{q}=\frac{\sigma^{2}}{\mu }+\frac{\mu }{N}$ and ${\hat{P}}_{\text{rel}}=\frac{\mu }{N\hat{q}}$ given a number of release sites *N* (Markram et al., 1997; Costa et al., 2015). The variance-mean analysis (also known as multiple-probability fluctuation analysis) is a slightly more advanced technique that relies on recording postsynaptic responses under different release probability conditions, which are typical set experimentally by varying the concentration of extracellular calcium. The relationship between the variance and the mean (i.e., μ and σ^2^ as above) under different release probabilities is then fitted to the parabolic function given by the binomial model (**Figure 3A**). This method estimates all three parameters (*N*, *P*_rel_, and *q*; see Lanore and Silver, 2016 for a detailed review on the topic). Because this method depends on having an accurate estimation of mean and variance of the postsynaptic responses, it requires relatively long and stable electrophysiological recordings under different conditions.

**Figure 2:**
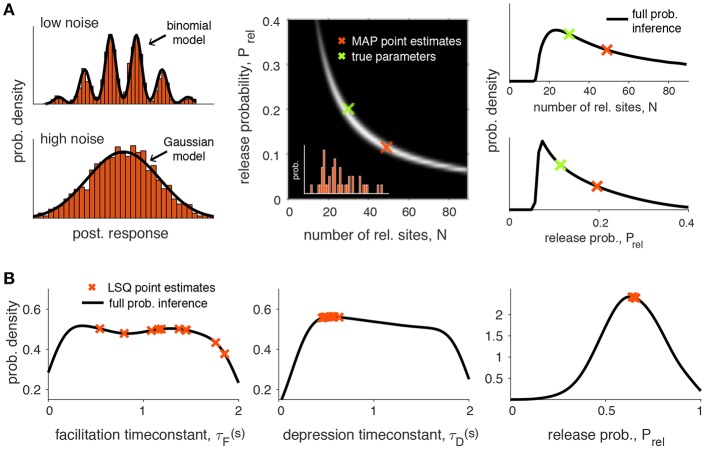
Identifiability of synaptic transmission parameters. **(A)** Identifiability issues of quantal release models. Left upper figure: Histogram of 2000 simulated postsynaptic responses with *N* = 5, *P*_rel_ = 0.5, *q* = 1, σ = 0.3. In this case it is possible to fit a binomial model. Left lower figure: same simulation, but for high noise (σ = 0.7). The quantal peaks (i.e., the parameter N) are not identifiable anymore if the recording noise is too high, and in this case a Gaussian model provides a better description of the synaptic responses. Middle panel: Pairwise posterior marginal for *N* and *P*_rel_ for a typical experimental case with 40 observations (simulated postsynaptic responses shown in inset) where the true parameters were *N* = 30, *P*_rel_ = 0.2 and *q* = 1 (green cross). The maximum a posterior (MAP) estimates is obtained for *N* = 49 and *P*_rel_ = 0.11 (red cross): as *N* and *p* are anticorrelated, the posterior is roughly the same over a long band were *N* and *p* can be substituted, leading to inference error for a small number of observations. Right panel: Marginal posterior for *N* and *P*_rel_ from the previous panel. **(B)** Identifiability issues of short-term synaptic plasticity models. Given experimental data it is often of interest to infer the synaptic parameters. Two main types of inference have been applied: point estimations where a single scalar is estimated for one or more parameters (red crosses) or full probabilistic inference, where the full probability density over the parameters is obtained (black line). This particular example was obtained by inferring the Tsodyks-Markram model with four parameters given short-term plasticity recordings between pyramidal cells in layer-5 visual cortex (see Costa et al., 2013 for more details, only three parameters are shown here for simplicity: τ_*F*_, τ_*D*_ and *P*_rel_). Point estimates were obtained using a standard least-square (LSQ) fitting method (simulated annealing). Full probabilistic inference was done using MCMC sampling following Costa et al. (2013) (see main text for more details). As demonstrated by Costa et al. (2013) the uncertainty over the parameters can be greatly reduced by using more informative protocols that cover a wider frequency range.

The binomial model described above may suffer from identifiability issues. For example, in the presence of a high level of noise it may not be possible to reliably separate the multiple peaks of the postsynaptic responses. In this case a simple Gaussian description of the synaptic responses may be preferable (Figure 2A). In addition, the methods described above also rely on point estimates which may lead to inaccurate conclusions due to correlations in the parameters (see Figure 2A for an example of such a case). A more principled approach to the problem that explicitly represents the uncertainty in the parameters should offer a better understanding of how well a particular model explains a given dataset (see section 3 for examples of this).

Building on earlier work (Turner and West, 1993), Bhumbra and Beato (2013) introduced a more principled quantal analysis method—Bayesian Quantal Analysis (BQA). This method applies Bayesian statistics which allows model inference to combine prior knowledge *P*(θ) over model parameters θ (e.g, θ = (*P*_*rel*_, *N, q*)) with the data likelihood *P*(*D*|θ) following Bayes' theorem as *P*(θ|*D*) ∝ *P*(θ)*P*(*D*|θ). In contrast to standard optimization methods, Bayesian inference explicitly models uncertainty over parameters given prior knowledge. Choosing the appropriate prior is an important step when developing Bayesian frameworks as it shapes the posterior distribution over parameters given by the likelihood. In BQA, the prior is used to integrate *a priori* knowledge about the synaptic release statistics (e.g., expected bounds), which simultaneously models the distributions of postsynaptic responses recorded under multiple release probabilities (independent of each other). This is in contrast with standard mean-variance analysis described above, which simply models the mean responses across different release probabilities. By incorporating prior information, this method improves the accuracy of parameter inference and, importantly, reduces the number of samples needed compared to the mean-variance analysis (from about 100 samples to about 60 samples). Therefore, this new method may be preferable in experimental conditions where long recordings are particularly challenging (see a more detailed comparison in Table 1).

**Table 1:** Comparison of different model-based approaches.

**Approach**	**Binomial**	**STP**	**Inference quality^h^**	**Experimental ease^h^**	**Algorithm complexity**
Mean-variance analysis^a^	✓	×	^**^	^*^(PSR)	O(M)
Bayesian quantal analysis^b^	✓	×	^***^	^**^(PSR)	O(MN)
Least-square STP fitting^c^	×	✓	^**^	^***^(PSR)	O(M)
Bayesian Gaussian-STP^d^	×	✓	^****^	^***^(PSR)	O(MS)
Binomial-STP^e^	✓	✓	^***^	^***^(PSR)	$O\left(MN^{4}\right)$
Bayesian binomial-STP^f^	✓	✓	^*****^	^***^(PSR)	$O\left(MN^{4}\right)$
Spike-based GLM^g^	×	✓	^*^	^****^(spikes)	O(M)

**Figure 3:**
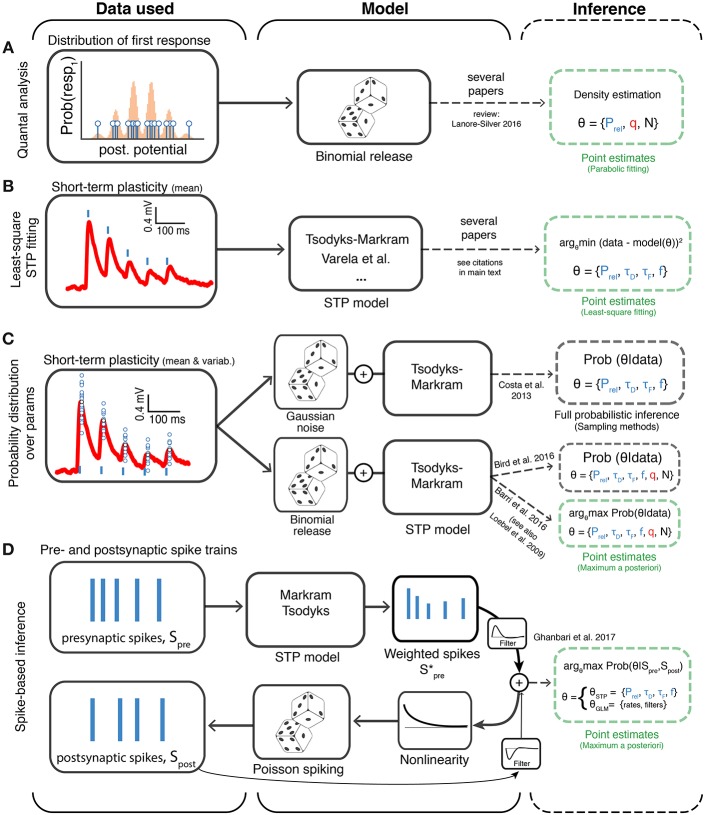
Different approaches to model-based inference of synaptic transmission. The different methods are organized based on the type of experimental data to which they are applied (first column), the model being assumed (second column) and the method of parameter inference (third column; dashed green and gray boxes indicate point estimate and full probabilistic inference, respectively). **(A)** Methods that use the variability of the first postsynaptic responses to infer binomial release statistics. **(B)** Methods that rely on multiple averaged responses to fit short-term plasticity (STP) models, which typically discard binomial release statistics. **(C)** Methods that directly consider both variability and multiple synaptic responses using probability theory to infer the synaptic transmission parameters. Here two variants have been explored: (i) a Bayesian framework where Gaussian noise is used to model the synaptic response variability (Costa et al., 2013) and (ii) a framework in which binomial release statistics are explicitly considered (Barri et al., 2016; Bird et al., 2016). The later has been explored using two variants: full inference (using sampling, Bird et al., 2016) and optimization methods (Barri et al., 2016). **(D)** Methods that work directly at the level of spike trains and try to infer short-term plasticity parameters. Ghanbari et al. (2017) introduced a new method based on generalized linear models (GLMs) to obtain point estimates of short-term plasticity models. *P*_rel_: Release probability; *q*: mean quantal amplitude; *N*: number of release sites; τ_*D*_: depression time constant; τ_*F*_: facilitation time constant; *f*: facilitation rate. Similarly to previous figures the mean postsynaptic responses are shown in red, spikes in blue (vertical lines) and small blue circles represent individual samples of postsynaptic responses.

*Note that the approaches that consider parameter uncertainty can be readily extended to Bayesian. In the O algorithm complexity analysis M refers to the number of data points, N to the number of release sites and S to the number of samples needed. Point estimate methods that obtain some measures of uncertainty of the parameters rely on getting multiple point estimates, whereas this comes naturally in full probabilistic methods (this is here reflected in the inference quality). The list of methods presented here is grouped into quantal methods (first two rows) and into STP models (last 5 rows) and then sorted by their publication date (earlier first). PSR: Postsynaptic responses. We use star-based ranking system for both inference quality and experimental ease, where one star means worse/harder*.

a *see Korn and Faber (1991), Lanore and Silver (2016), and Figure 3A*.

b *see Bhumbra and Beato (2013) and Figure 3A*.

c *see for example Markram et al. (1998), Le Bé and Markram (2006), Markram (2006), Wang et al. (2006), Rinaldi et al. (2008), Ramaswamy et al. (2012), Testa-Silva et al. (2012), Romani et al. (2013) and Figure 3B*.

d *see Costa et al. (2013) and Figure 3C*.

e *see Loebel et al. (2009), Barri et al. (2016), and Figure 3C*.

f *see Bird et al. (2016) and Figure 3C*.

g *see Ghanbari et al. (2017) and Figure 3D*.

h *Note that this ranking is subjective and based purely on our experience with these methods*.

## 3. Inference of Short-Term Plasticity

Postsynaptic responses are dynamic—the peak response amplitude depends not only on the quantal parameters, but also on previous activity. If the presynaptic neuron fires in quick succession, the released vesicles are not given enough time to be recycled, which leads to less vesicles available for release. As a consequence synaptic responses become weaker, also known as *short-term depression* (Figure 1A) and such recovery rates are often modeled with an exponential with timeconstant τ_*D*_. At the same time the presynaptic calcium levels can increase with every consecutive spike, which may lead to an increase in the postsynaptic response rather than a decrease—this is known as *short-term facilitation*.

### 3.1. Deterministic Models of Short-Term Plasticity

A number of deterministic short-term plasticity models have been proposed that characterize the dynamic properties of synaptic transmission (for a review on STP models see Hennig, 2013). These models capture STP data relatively well, and thus may enable us to uncover how STP may be regulated under different conditions.

The parameters of these models are commonly fit using least-squares optimization to obtain a single set of parameters (point estimates) where the goal is to find the best (or at least a good) set of parameters that captures a given experimental dataset (Markram et al., 1998; Le Bé and Markram, 2006; Markram, 2006; Wang et al., 2006; Rinaldi et al., 2008; Ramaswamy et al., 2012; Testa-Silva et al., 2012; Romani et al., 2013) (Figure 3B).

However, estimating parameters of STP models poses a challenge. Similar to the issues highlighted above for binomial models, in most STP models different parameter sets produce model outputs that follow the observed data equally well (Figure 2B; Costa et al., 2013). The existence of these multiple plausible solutions opens problems when relying on point estimates to draw conclusions about the underlying biological mechanisms. Therefore, it is important to also consider the uncertainty of the parameter estimation. Unlike single point estimate approaches, full probabilistic inference naturally captures parameter uncertainty, which enables a more comprehensive model comparison (e.g., Akaike Information Criterion, Bayesian Information Criterion or Bayes factor). Note that this can also be in principle obtained using sensitivity or cross-validation analysis when using standard fitting methods (Varela et al., 1997; Tennøe et al., 2018), but as highlighted in Figure 2B these methods may not provide a complete picture of the parameter landscape. One form of probabilistic inference is full Bayesian inference where, similar to the BQA approach, we aim to obtain the posterior distribution of STP parameters given experimentally observed data.

Costa et al. (2013) introduced the first Bayesian inference framework of STP models (Bayesian Gaussian-STP; Figure 3C; Table 1). In this work the authors modeled the mean postsynaptic peak responses using the Tsodyks-Markram STP model to account for the dynamic properties of the synapse (Tsodyks and Markram, 1997; Markram et al., 1998). The Tsodyks-Markram STP model is a commonly used model built around the synaptic dynamics discussed above. In order to capture the variability of synaptic responses, Costa et al. (2013) used a Gaussian approximation as the likelihood and a flat (uninformative) prior with reasonable bounds over the parameters. Calculating the posterior exactly is often intractable due to complex likelihoods and intractable normalizing constants. Instead, Costa et al. (2013) obtained the posterior distribution *P*(θ|*D*) via sampling using a Markov Chain Monte Carlo (MCMC) algorithm. MCMC methods rely on constructing a Markov chain^2^ that should converge to the desired probability distribution in the equilibrium (i.e., after long enough observations).

This method was used to study the parameter uncertainty given datasets obtained with common experimental protocols, which are typically based on regular spike trains. The posterior distributions revealed that some of the parameters from the Tsodyks-Markram STP models were poorly constrained by such experimental protocols (Figure 2B). This observation led to the proposal of new and irregular experimental protocols that span a broader stimulation frequency range and result in substantially reduced uncertainty over the parameter values. Such protocols not only lead to reduced uncertainty, but can also be more easily applied in realistic and natural conditions (Dobrunz and Stevens, 1999).

Furthermore, obtaining the posterior distribution helps to understand the dependencies between parameters, which is not straightforward using traditional fitting methods. For example, in Stone et al. (2014), the authors used an MCMC method to obtain the posterior distribution over the parameters (similar to Costa et al., 2013) allowing the authors to highlight two strongly correlated parameters. Importantly, the identification of this correlation led to a reparameterization of the model which improved parameter inference. Therefore, obtaining the posterior distribution over the parameters makes it possible to characterize their uncertainty and explore possible dependencies between parameters. Such MCMC methods are relatively efficient as long as the model can be computed efficiently (up to a few seconds) and the number of STP parameters remains relatively low (less than a few dozens).

### 3.2. Stochastic Short-Term Plasticity Models

There are two important limitations of relying on deterministic STP models. First, the optimization depends on an accurate estimation of the mean synaptic responses. As mentioned above, this requires a high number of trials, which is experimentally challenging (see Table 1). Second, by only considering averages these methods ignore the correlations between postsynaptic peaks, yet these correlations may provide valuable information to accurately infer the synaptic properties.

A couple of recent studies introduced methods that incorporate correlations between postsynaptic responses in the inference of STP parameters. These methods allow the extraction of both quantal and dynamic parameters of synaptic transmission from trains of postsynaptic responses without the requirement of averaging over multiple sweeps (Loebel et al., 2009; Barri et al., 2016; Bird et al., 2016). These studies implemented stochastic models of synaptic transmission by combining phenomenological Tsodyks-Markram STP models with binomial models of vesicular release and replenishment. The probability of vesicle release is derived from a Tsodyks-Markram model and the vesicle replenishment probability is modeled with a Poisson process controlled by a depression time constant parameter τ_*D*_. The quantal size of the postsynaptic response evoked by each vesicle can be approximated by either a gamma distribution in Bird et al. (2016), or an inverse Gaussian distribution in Barri et al. (2016), and the total amplitude is modeled as a linear combination of these distributions. The choice of these distributions is motivated by the fact that the quantal amplitude distribution is positively skewed, a feature that can not be captured by a Gaussian distribution.

The full formulation of the stochastic STP model can be used to define the likelihood of the observed data given the model, *P*(*D*|θ). The stochasticity of the model introduces correlations between peaks in the train and these correlations pose the main difficulty in the likelihood calculation. In particular, because the amplitude of the postsynaptic response is dependent on the number of released vesicles. As discussed in Barri et al. (2016), if the likelihood is to be formulated using the probability distribution of released vesicles, the number of terms in the calculation would grow exponentially. This becomes a permutation with repetition problem, in order to account for correlations of released vesicles the number of terms in the calculation would grow as (*N* + 1)^*K*^ with *N* being the number of release sites and *K* corresponding to the number of spikes in the train. To make the calculation more efficient, in both studies the likelihood function is formulated in terms of the probability distributions of the release sites before and after a spike (rather than continuously), which fully captures the state of the system.

These two studies apply different strategies to obtain point estimates from the likelihood. Barri et al. (2016) uses an expectation-maximization algorithm (referred to as Binomial-STP in Table 1; see also Loebel et al., 2009), while Bird et al. (2016) uses MCMC sampling and flat priors (referred to as Bayesian binomial-STP in Table 1). While both methods return a point estimate of the parameter set that maximizes the likelihood function, only the sampling approach approximates the joint likelihood distribution of the parameters. As discussed above, by obtaining the full likelihood, not just a point estimate, Bird et al. (2016) explicitly quantifies the uncertainty over the parameters, and the full likelihood density (or posterior) can be analyzed. Moreover, it also allows for correlations between the distributions over the parameters to be studied.

The main features of these approaches are (i) accounting for correlations between subsequent postsynaptic responses and (ii) using individual postsynaptic traces for fitting the models, which offers theoretical and practical advantages. Interestingly, both Barri et al. (2016) and Bird et al. (2016) report that considering correlations during inference yields estimates of synaptic parameters that are more accurate and require less sweeps when compared to ignoring correlations. This means that the experimental protocols can be shorter, hence making these inference methods particularly attractive for experiments *in vivo*.

## 4. Toward Inference of Synaptic Transmission *in vivo*

Recent developments have started to raise the possibility of accurately inferring synaptic transmission properties *in vivo*. One way to tackle this problem is to perform whole-cell recordings *in vivo* while stimulating the presynaptic neurons (or presenting a stimuli) (Costa et al., 2015; Pala and Petersen, 2015; Sedigh-Sarvestani et al., 2019). This is a valuable approach that is enabling the community to confirm previous *in vitro* results *in vivo*. For example, Costa et al. (2015) applied binomial-based estimation methods typically used in slices to *in vivo* data, and obtained results consistent with both modeling predictions and slice data. Puggioni et al. (2017) and Latimer et al. (2018) introduced new statistical methods with some success in inferring synaptic conductances from *in vivo* intracellular recordings and spike trains, respectively. However, these methods were not developed to estimate quantal or synaptic dynamics properties. In order to test how such synaptic features are shaped in more natural conditions across different brain regions new methods are required that can operate on the growing imaging-based or spike-based datasets.

Detecting synaptic connections from spikes alone is challenging. Even in the case of simple monosynaptic connections this is not straightforward (Fetz et al., 1991), but there have been recent successful attempts (English et al., 2017). One of the key difficulties in inferring synaptic parameters from spikes is that several non-synaptic variables can have an impact on the spiking statistics (Stevenson et al., 2008). For example, when a presynaptic neuron fires at high frequencies one would expect a reduction in the firing rate of the postsynaptic neuron due to short-term depression at their synaptic connections, but a similar effect can also be mediated by postsynaptic neuron-wide adaptation mechanisms (Brette and Gerstner, 2005).

A first attempt at tackling this problem has recently been put forward (Ghanbari et al., 2017). In this framework, the authors extended a generalized linear model to infer both neuronal and STP parameters directly from spike-trains (Figure 3D, referred to as spike-based GLM in Table 1). Interestingly, using their framework Ghanbari et al. (2017) showed that in a reduced system—a single postsynaptic neuron in slices with simulated inputs—postsynaptic adaptation can be distinguished from short-term depression as they are predominantly correlated with pre- and postsynaptic firing rates, respectively. More recently the same authors (Ghanbari et al., 2018) went further and used their framework to show that functional connectivity with STP may explain the diversity of activity patterns observed *in vivo* between different brain areas. However, for these approaches to provide accurate estimates of synaptic transmission properties (Table 1) *in vivo* many other factors need to be considered in future work, such as network dynamics, cell-type specificity and dendritic integration.

As highlighted above (section 3), inferring synaptic parameters using naturalistic conditions (e.g., spike patterns) not only is likely to give more precise estimates of synaptic parameters, but also insights into which synaptic transmission properties are relevant in behaving animals (Dobrunz and Stevens, 1999; Isaac et al., 2009).

## 5. Discussion

In this review we have provided an overview of standard methods and recent developments of model-based inference of synaptic transmission. We started out by reviewing methods that rely on the binomial statistics of the first postsynaptic response alone and moved on to methods that consider the dynamics of consecutive synaptic responses (short-term plasticity) and their statistical properties. Historically, inference methods have mostly focused on point estimations, which give a biased interpretation of synaptic data (Costa et al., 2013). More recent developments have focused on full probabilistic inference, thus providing a more comprehensive picture on the most likely synaptic transmission parameters (Bhumbra and Beato, 2013; Costa et al., 2013; Bird et al., 2016).

One research direction that should improve the inference quality of the short-term plasticity parameters is to optimize the experimental protocol, namely the timings of the presynaptic action potentials. The stimulation protocol needs to be within some acceptable range (a too high stimulation frequency would induce long-term plasticity and thereby violate the stationarity assumption). However, within such a range, there is a lot of freedom that can be exploited to improve the quality of the parameter estimates. For example, Costa et al. (2013) explored a few different protocols (regular spike trains, regular spike train + recovery spike(s) or Poisson spike trains). It would be important to systematically study the space of protocols and determine which ones are the most informative. Pushing this idea even further, it would be interesting to design a closed-loop inference scheme such that after each spike and its subsequent postsynaptic response, the algorithm determines the best interval for the next spike that is maximally informative about the synaptic parameters.

In this review, we have not covered some other properties that are of interest. One that has received attention recently is the inference of the size of the presynaptic readily-releasable vesicle pool (Abrahamsson et al., 2017; Barros-Zulaica et al., 2019). Additionally, we have focused on the binomial release model, but many synapses require different release probabilities and quantal amplitudes across release sites, which is better captured by multinomial statistics (Walmsley et al., 1988; Lanore and Silver, 2016). There are several other important aspects of synaptic transmissoin not considered here, such as constrains on trial-to-trial quantal variability (Kullmann, 1993), STP models that also account for changes in quantal amplitude (Scheuss et al., 2002), frequency-dependent recovery rates in STP (Fuhrmann et al., 2004), and release-independent short-term depression (Bellingham and Walmsley, 1999; Fuhrmann et al., 2004). In future work, it would be important to understand how the developments reviewed here can also consider and be used to better understand these finer aspects of synaptic transmission.

There have been remarkable developments in measuring synaptic properties with high temporal and spatial resolution (Rey et al., 2015; Tang et al., 2016). Of particular interest are recent advances in ultrafast optical glutamate sensors, which are enabling measurements of synaptic release with high accuracy (Helassa et al., 2018). These developments, when coupled with the statistical inference frameworks reviewed here (Costa et al., 2013; Bird et al., 2016; Ghanbari et al., 2017, but see also Soares et al., 2019), raise the possibility of accurate optical estimation of synaptic transmission properties in awake behaving animals.

Finally, there has been a recent surge in new and exciting large-scale recordings, such as voltage and calcium imaging (Piatkevich et al., 2019), multi-patch recordings (Peng et al., 2019) and multi-electrode spike recordings (Jun et al., 2017). With such methods at hand now is the right time to start asking questions that bridge systems neuroscience and synaptic transmission properties. By building on initial studies on how synapses are shaped by naturalistic spike-trains (Dobrunz and Stevens, 1999; Isaac et al., 2009), this body of work opens the possibility of inferring quantal and dynamic properties of synapses over multiple brain areas as animals learn a particular task.

Taken together these novel inference and experimental methods open the possibility of testing different theories put forward for the role of synaptic transmission in learning and memory (Pfister et al., 2010; Costa et al., 2015, 2017; Llera-Montero et al., 2019), but also their impact in pathological states (Jackson et al., 2017).

## Author Contributions

OB, CG, and RC generated the figures. OB, CG, A-LS, DJ, MM, AB, CH, J-PP, and RC wrote the manuscript.

### Conflict of Interest Statement

The authors declare that the research was conducted in the absence of any commercial or financial relationships that could be construed as a potential conflict of interest.

## Footnotes

^1^The binomial release model makes a few assumptions, namely that each site releases vesicles independently and that *P*_rel_ is the same across different release sites.

^2^A Markov chain represents a probabilistic transition between states, in which a given transition depends only on the previous state.
